# *Fluviispira vulneris* sp. nov., isolated from human wound secretions

**DOI:** 10.1007/s10482-023-01883-4

**Published:** 2023-09-29

**Authors:** Peijuan Tang, Na Peng, Pengwen Ouyang, Sheng Long, Zhenhua Wei, Xingchun Chen, Pinghua Qu, Liangyi Xie

**Affiliations:** 1 Department of Clinical Laboratory, Hunan Province People’s Hospital, The First Affiliated Hospital of Hunan Normal University, Changsha, 410005 People’s Republic of China; 2 People’s Hospital of Mashan, Nanning, 530699 Guangxi People’s Republic of China; 3 The People’s Hospital of Guangxi Zhuang Autonomous Region, Nanning, 530022 Guangxi People’s Republic of China; 4 Department of Clinical Laboratory, The Second Clinical College of Guangzhou University of Chinese Medicine, Guangdong Provincial Hospital of Traditional Chinese Medicine, Guangzhou, 510006 People’s Republic of China

**Keywords:** *Fluviispira vulneris* sp. nov., Wound infections, 16S rRNA, Polyphasic taxonomy

## Abstract

Human infections by environmental bacteria is becoming an increasing problem and has become a matter of great concern due to the adverse effects worldwide. In this study, we reported a new environmental pathogen. Isolate GX5518^T^ was a novel Gram-negative, aerobic, non-motile, pleomorphic and red-pigmented bacterium, was isolated from human wound secretions (GuangXi, People’s Republic of China). Growth occurred at pH 6.0–8.0 (optimum, pH 7.0) and 10–37 °C (optimum, 28–32 °C) with 0–1.5% (w/v) NaCl in R2A agar. Comparative analysis of the 16S rRNA gene sequences revealed that isolate GX5518^T^ was closely related to *Fluviispira sanaruensis* JCM 31447^T^ (99.73%) and *Fluviispira multicolorata* 33A1-SZDP^T^ (98.49%). However, the estimated ANI values of the isolate GX5518^T^ compared to the *F. sanaruensis* JCM 31447^T^ and *F. multicolorata* 33A1-SZDP^T^ were 88.67% and 77.35%, respectively. The estimated dDDH, ANI and AAI values between isolate GX5518^T^ and its closely related strains were below the threshold values generally considered for recognizing a new species. The genome size was 3.6 Mbp and the DNA G + C content was 33.1%. The predominant fatty acids (> 5%) in GX5518^T^ cells were iso-C_15:0_, C_16:0_, C_17:0_, C_17:1_ *ω*8*c* and C_16:1_ *ω*7*c*/C_16:1_ *ω*6*c*. The major menaquinone was MK-8 (86.9%). The polar lipids were phosphatidylethanolamine (PE), phosphatidylglycerol (PG) and three unknown lipids (L1-3). The chemical composition was different from that of the *F. sanaruensis* JCM 31447^T^. Comparative genomics analysis between isolate GX5518^T^ and its related strains revealed that there were a number of genes involved in resistance to antibiotics and toxic compounds in isolate GX5518^T^, which were responsible for the copper homeostasis, cobalt-zinc-cadmium resistance, resistance to fluoroquinolones, and zinc resistance. Based on the phenotypic, chemotaxonomic, and genomic analyses, isolate GX5518^T^ (= CGMCC 1.18685^T^ = KCTC 82149^T^) represents a novel species of the genus *Fluviispira*, for which the name *Fluviispira vulneris* sp. nov. is proposed.

**Supplementary Information:**

The online version contains supplementary material available at 10.1007/s10482-023-01883-4.

## Introduction

The genus *Fluviispira* (Pitt et al. 2020), a member of the family *Silvanigrellaceae* (Hahn et al. 2017) within the order *Silvanigrellales*, the class *Oligoflexia* and the phylum *Bdellovibrionota*, is generally characterized as Gram-negative, aerobic, motile, non-spore forming, oxidase negative and pleomorphic. At the time that the strain was isolated, the genus only consists of one validly named species, *F. multicolorata* (Pitt et al. 2020). Subsequently, *F. sanaruensis* was described by Maejima et al. (2021) as a novel member of the genus *Fluviispira. F. multicolorata* grown on NSY agar form purple-pigmented, circular colonies but liquid cultures appear either purple, grey or orange, which was isolated from a small creek in Austria. *Fluviispira sanaruensis* produce a salmon pink on R2A agar, which was isolated from brackish lake water sampled at Lake Sanaru in Japan. The above strains were all isolated from freshwater samples in different countries and no cases of human infections. It is worth mentioning that “*Pigmentibacter rube*r” (Peng et al. 2021) was isolated from a blood specimen of a patient after a drowning accident in our country as a novel genus and species of the family *Silvanigrellaceae*, its emergence was the first case of human infections caused by the family *Silvanigrellaceae.*

In 2019, an 86-year-old man was admitted to the first affiliated hospital of Hunan Normal University with “stone-smashed right calf pain and bleeding for 4 h”. Inflammatory indicators such as leucocyte (WBC), neutrophilic granulocyte percentage (NEUT%), procalcitonin (PCT) and C-Reactive Protein (CRP) increased. Necrotic tissue debridement + VSD negative pressure drainage operation was performed, intraoperative tissue and secretions were sent for bacterial culture, and a novel Gram-negative, aerobic, pleomorphic, red-pigmented isolate was reported, designed GX5518^T^.

The current study aimed to determine the precise taxonomic position of isolate GX5518^T^ by using a polyphasic taxonomic approach.

## Materials and methods

### Isolation and cultivation

Specimens for bacterial culture were obtained from wound tissue and secretion of a patient with bleeding from a right leg injury in Nanning, Guangxi Province, PR China. A Gram-negative, aerobic, pleomorphic, red-pigmented isolate, designed as GX5518^T^, was isolated as potential pathogen with dominant from Columbia blood agar (bioMérieux) and ordinary chocolate plate (bioMérieux) at 37 °C for 24 h. Purified cultures were maintained in glycerol suspensions (30%, v/v) with 2% blood at − 80 °C for further polyphasic taxonomy investigation. Isolate was also deposited in the Korean Collection for Type Cultures (KCTC 82149^T^) and China general microbiology culture collection center (CGMCC 1.18685^T^). Two type strains, *F. sanaruensis* JCM31447^T^ and *F. multicolorata* 33A1-SZDP^T^, were chosen as reference type strains and were cultured under the same experimental conditions for comparative studies.

### Morphological, physiological and biochemical characterization

Cell morphology of isolate GX5518^T^ was observed using Optical microscope (BH-2, Olympus) and transmission electron microscopy (JEM1200, JEOL) (Ming et al. 2012) with the exponential-phase cells negatively stained with phosphotungstic acid. Motility was examined using the hanging-drop technique and on semi-solid R2A soft agar (containing 0.3% agar) (Jeong et al. 2020). Gram-staining was tested by using the Gram-stain kit (BaSo, China) and was confirmed using the KOH lysis test (Krishna and Gole 2017).

To determine the optimal growth conditions, growth tests were performed on different media, including Columbia blood agar, chocolate agar, MacConkey agar, Lysogeny broth (LB) agar, Mueller–Hinton (MH) agar, defibrinated sheep blood Mueller–Hinton (Blood-MH) agar, R2A agar, 1/2 R2A agar, Blood-R2A agar, tryptone soya (TSA) agar, Blood-TSA agar, 2216E agar, brain heart infusion (BHI) agar and BCYEα agar at 35 °C under air atmosphere and 5% CO_2_ atmosphere, respectively. Explored the optimal growth temperature (5, 10, 15, 20, 25, 28, 32, 35, 37, 40 and 42 °C) and the optimal pH range (pH 4.0–10.0, at intervals of 1 pH unit) on Blood-R2A agar at 35 °C under ordinary air atmosphere. The pH values of the Blood-R2A agar were adjusted by using citrate buffer (for pH 4.0–5.0), phosphate buffer (for pH 6.0–7.0), Tris buffer (for pH 8.0–9.0) and Na_2_CO_3_/NaHCO_3_ buffer (for pH 10.0). NaCl tolerance (0.5–5%, w/v, with an interval of 0.5%) were determined in Blood-R2A agar at 35 °C. Anaerobic growth was tested using anaerobic bags (bioMérieux, France).

Oxidase activity was tested by using 1% (w/v) tetramethyl-*p*-phenylenediamine (Kovacs 1956). Catalase activity was determined by using 3% (v/v) hydrogen peroxide (Jeffries et al. 1957). Other physiological and biochemical characteristics and enzyme activities were determined using API 20 NE and API ZYM test-strip systems (bioMérieux, France) at 35 °C according to the manufacturers’ instructions and *F. sanaruensis* JCM 31447^T^ was used as control. Because of the isolate GX5518^T^ grew slowly on the MH agar, antimicrobial susceptibility profile was assessed by determining minimum inhibitory concentrations (MICs) using a commercial assay (Blood-MH agar, E-test) according to manufacturer's instructions.

### Chemotaxonomic analysis

The fatty acids profiles of isolate GX5518^T^ and *F. sanaruensis* JCM 31447^T^ were analysed using cells grown on Blood-R2A agar for incubated at 35 °C for 72 h until bacterial cultures reached the exponential phase. Acids methyl esters were extracted and identified by using gas chromatography (6890, Agilent) and the MIDI Sherlock Microbial Identification System (MIDI) system (Sherlock version 6.3; MIDI database: TSBA6) following the manufacturer’s instructions. (Sasser 1990; Tindall 1990; Collins et al. 1982). Polar lipids of the isolates were extracted and analysed by using two-dimensional thin-layer chromatography (2D-TLC). The first phase was chloroform: methanol: distilled water = 65:25:4 (v/v), and the second phase was chloroform: glacial acetic acid: methanol: distilled water = 80:18:12:5 (v/v). (Minnikin et al. 1979). Total lipids were detected by phosphomolybdate and amino lipids, phospholipids and lecithin were detected by ninhydrin, D reagent and molybdenum blue respectively. Menaquinone were extracted and purified from lyophilized cells, and identified by using an HPLC (LC-20AT, Shimadzu) (Goodfellow et al. 1980).

### Phylogenic and genomic analysis

The genomic DNA of the isolate GX5518^T^ was extracted and purified using the method as described previously (Weisburg et al. 1991). 16S rRNA gene was amplified using the bacterial universal primers of 27F (5′-AGAGTTTGATCCTGGCTCAG-3′) and 1492R (5′-GGGTTACCTTGTTACGACTT-3′) previously described (Frank et al. 2008). PCR amplification parameters were: 95 °C, pre-denaturation, 5 min; 35 cycles (95 °C, denaturation, 30 s; 53 °C, annealing, 30 s; 72 °C, extension, 1 min); 72 °C, extension, 7 min (Ludwig 2007). The amplified products were purified using a PCR purification kit (Takara). The sequenced data were assembled using the DNAMAN version 8 software. Chromas version 1.62 software was applied to analyse the sequencing profiles. The 16S rRNA gene sequence of GX5518^T^ was compared with other sequences on BLAST database (https://blast.ncbi.nlm.nih.gov/) (Johnson et al. 2008) and EZbioucloud database (http://www.ezbiocloud.net/) (Yoon et al. 2017) and downloaded the adjacent gene sequences. Multiple alignments with sequences by using Clustal_W (Larkin et al. 2007). Phylogenetic tree reconstructions were performed based on neighbor-joining (NJ) (Saitou and Nei 1987), maximum-likelihood (ML) (Felsenstein 1981) and maximum-parsimony (MP) (Fitch 1971) algorithms by using MEGA version 11.0 (Tamura et al. 2021). Evolutionary distances in the NJ and ML dendrograms were calculated by the Kimura two-parameter model (Kimura 1980) and used the bootstrap resampling methods based on 1000 replications (Felsenstein 1985). *Geobacter metallireducens* DSM 7210^T^ (NR_025895) was used as the outgroup.

The genomic DNA of isolate GX5518^T^ were sequenced using Illumina Hiseq platform by Novogene Company (Beijing, China). The raw data were filtered by readfq (version 10) and assembled by using the software SOAP de novo (Bankevich et al. 2012). Genome completeness and contamination of the sequences were estimated using CheckM (Thompson et al. 2013). For generation of a phylogenomic tree, the genome sequences of the related type strains with the isolate GX5518^T^ were retrieved from the NCBI database and the phylogenomic tree based on the core genes was built by using UBCG (Up-to-date Bacterial Core Gene) (Na et al. 2018) with default settings.

To determine the genomic relatedness of isolate GX5518^T^ and the related type strains available in public databases, the average nucleotide identity (ANI) based on blastn (ANIb) and MUMmer (ANIm) between the novel strain and closely related type strains were calculated by using the online JSpeciesWS (http://jspecies.ribohost.com/jspeciesws/) (Richter et al. 2016; Kim et al. 2021). The average amino acid identity (AAI) values were determined by using CompareM software (https://github.com/dparks1134/CompareM) (Palmer et al. 2020; Qu et al. 2021; Zheng et al. 2020). The digital DNA-DNA hybridizationd (DDH) values were calculated using the DSMZ Genome-to-Genome Distance Calculator (GGDC) version 3.0 (http://ggdc.dsmz.de/ggdc.php) (Meier-Kolthoff et al. 2022). To further understand the metabolic features of the novel strain and its close relatives, annotation was performed by using the Rapid Annotations in the Subsystem Technology (RAST) server (http://rast.nmpdr.org/) (Aziz et al. 2008; Overbeek et al. 2014). The Cluster of Orthologous Groups (COGs) of proteins was used to analyse functional classification of the protein-coding genes of isolate GX5518^T^ by using server database called evolutionary genealogy of genes: Nonsupervised Orthologus Groups (http://eggnog5.embl.de/#/app/home) (Huerta-Cepas et al. 2019). The antibiotic resistance genes and virulence genes were evaluated using the Comprehensive Antibiotic Research Database (CARD) (https://card.mcmaster.ca/analyze/rgi) (Alcock et al. 2020) and Virulence Factors Database (VFDB) (http://www.mgc.ac.cn/cgi-bin/VFs/v5/main.cgi) (Chen et al. 2005) to evaluate the potential pathogenicity of the strains.

### Animal experiments and histopathological analysis

To assess the virulence of the isolate GX5518^T^, the animal experiments were carried out on 64 SPF-grade KM male and female mice, weighting (18 ± 2) g, which were purchased from Slack Jing da (Hunan) Laboratory Animal Technology Co., Ltd. The feeding temperature of mice is 18–25 °C, and the humidity is 50–70%. Mice were acclimated for a week before the experiment. The purified culture was made into a bacterial solution with a turbidity of 3.0 MCF using a 0.9% NaCl solution, and the concentration was calibrated using a turbidimeter (bioMérieux). The bacterial suspension was diluted to 8 gradient concentrations using the doubling dilution method. Diluted bacterial suspension was injected into mice by intraperitoneal injection, 8 mice in each group, 8 groups in total, and the injection volume was 0.02 ml/g. The mice in the negative control group were injected with the same amount of 0.9% sodium chloride. Calculate LD50 by the modified Cole's method (Yang et al. 2020). Traits, body weight and mortality of the mice were monitored and recorded daily for 7 days. The tissues, including hearts, livers, spleens, lungs, and kidneys, were collected, for tissue sections and HE staining, following the pathological procedures (Qian et al. 2020). Ethical permission for animal experiments is provided in the Supplementary Annex.

## Results and discussion

### Morphological, biochemical, and physiological characterization

**Fig. 1:**
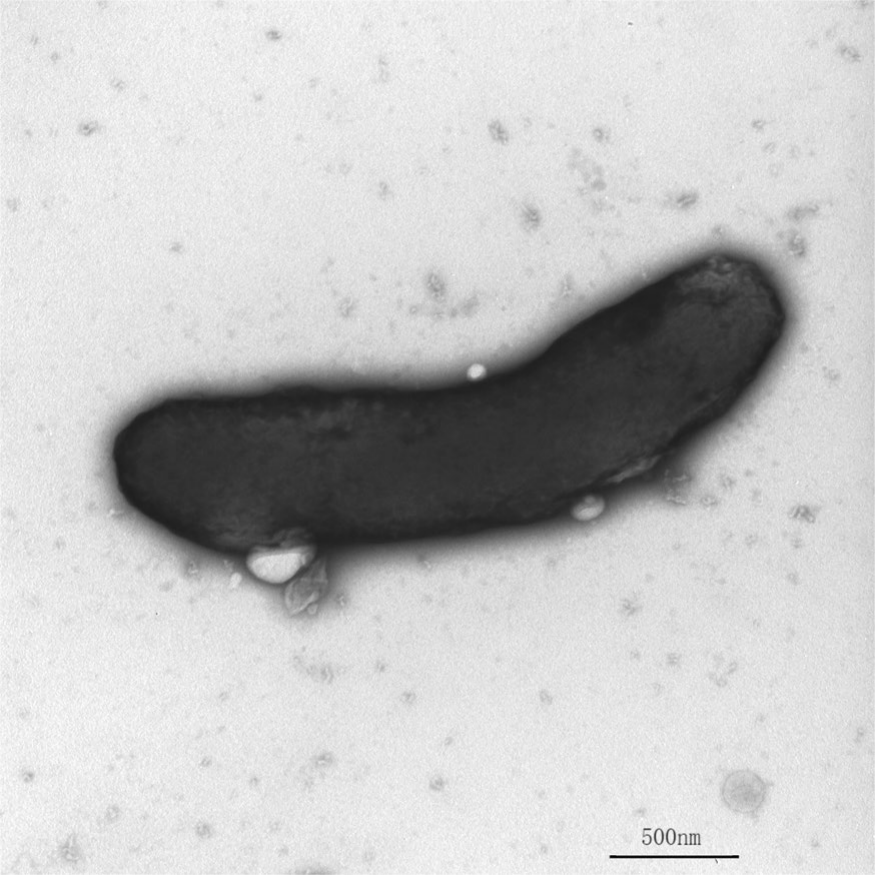
Transmission electron micrograph of isolate GX5518^T^ from cultures grown on R2A agar for 3 days at 32 ℃. Bars 5 µm

Isolate GX5518^T^ was observed to form red-pigmentation, round, smooth, convex colonies after 72 h of incubation on R2A agar at 35 °C with a 5% CO_2_ atmosphere. The cell was Gram-negative, aerobic, motile, and pleomorphic (Figs. S1–S2). Under the transmission electron microscope, the main cell shapes were rod, some were spirals (0.6–1.0 μm × 1.8–3.2 μm, Fig. 1). The semi-solid agar dynamic test was negative. Isolate GX5518^T^ could grow on Columbia blood agar, chocolate agar, R2A agar, Blood-R2A agar, BCYEα agar, Blood-TSA agar and Blood-MH agar. It was able to grow at 10–37 °C (optimum, 28–32 °C), pH 6.0–8.0 (optimum, pH 7.0) and in the presence of 0–1.5% (w/v) NaCl (optimally without NaCl), in which the salt concentration reached 1.5%, had a certain antibacterial effect. The formation of bubbles in 3% (v/v) hydrogen peroxide and the lack of blue colour in a colony after oxidation with 1% (w/v) tetramethyl-*p*-phenylenediamine showed that the isolate was catalase-positive and oxidase-negative, respectively. Different characteristics of isolate GX5518^T^ and its related species were shown in Table 1. In API 20NE and API ZYM test strips, isolate GX5518^T^ was positive for alkaline phosphatase and acid phosphatase, and weakly positive for esterase (C4), esterase lipase (C8) and naphthol-AS-BI-phosphohydrolase, negative for oxidase and could not reduce nitrate or produce indole, and was not capable assimilation of arginine dihydrolase, urease, lipase (C14), cystine arylamidase, *α*-mannosidase, *α*-chymotrypsin, *α*-galactosidase, *β*-galactosidase, *β*-glucuronidase, *α*-glucosidase, *β*-glucosidase, *α*-mannosidase, *β*-fucosidase, capric acid, adipic acid, malate, trisodium citrate and phenylacetic acid, which showed similar results as those from closely related strains, *F. sanaruensis* JCM 31447^T^, *F. multicolorata* 33A1-SZDP^T^, “*P. ruber*” KCTC 72920^T^ and *S. aquatica* DSM 23856^T^. However, it could be clearly distinguished from them in catalase, fermentation (glucose), esculin, gelatin hydrolysis,, assimilation of glucose, arabinose, mannose, mannitol, *N*-acetyl-glucosamine, maltose, potassium gluconate and hydrolyses of leucine arylamidase, valine arylamidase, trypsin arylamidase, *N*-acetyl-*β*-glucosaminase.

**Table 1:** Differential characteristics of isolate GX5518^T^ and its related species

Characteristics	1	2	3	4	5
Oxygen requirement	Aerobic	Aerobic	Aerobic	Aerobic	Aerobic
Gram stain	Negative	Negative	Negative	Negative	Negative
Pigmentation (agar plates)	Pink	Salmon pink	Purple	Red	Red
Cell morphology	Pleomorphic	Pleomorphic*	Pleomorphic*	Pleomorphic	Pleomorphic*
Temperature range for growth (℃)	10–37	10–34*	6–34*	15–37	10–32*
NaCl tolerance (%, w/v)	0–1.5	0–1.0*	1.0–1.2*	0–1.5	0–1.0*
pH	6–8	7–8*	ND*	6–8	7–8*
Oxidase	−	−	−	−	−
Catalase	+	+	−	w	w
Fermentation (glucose)	−	w	−	−	−
Esculin	w	w	−	+	−
Gelatin hydrolysis	−	+	−	+	−
β-Galactosidase	−	−	−	+	−
Glucose	−	−	−	+	−
Arabinose	−	−	−	−	w
Mannose	−	−	−	+	−
Mannitol	−	−	−	+	−
*N*-acetyl-glucosamine	−	−	−	w	−
Maltose	−	−	−	+	−
Potassium gluconate	−	−	−	w	−
Alkaline phosphatases	+	+	+	+	+
Esterase (C4)	w	w	w	w	w
Esterase lipase (C8)	w	w	w	w	w
Leucine arylamidase	+	−	−	+	+
Valine arylamidase	w	−	−	w	w
Trypsin arylamidase	−	−	−	w	−
Acid phosphatases	+	+	+	+	+
Naphthol-AS-BI-phosphohydrolase	w	+	+	w	w
*N*-acetyl-β-glucosaminase	w	+	+	−	−
Main polar lipids	PE and PG	PE and PG*	PE and PG*	PE and PG	PE and PG*
Main fatty acids	iso-C_15:0_, C_17:0_ and C_16:0_	iso-C_15:0_, C_16:0_ and summed feature 3	iso-C_15:0_ and C_17: 1_ ω8c*	iso-C_15:0_ and C_16:0_	iso-C_15:0_, anteiso-C_15:0_, C_16:0_ and feature 3*
Menaquinone	MK-8	ND*	MK-8*	MK-8	ND*
G + C content (mol%)	33.1	33.7	32.2	29.6	32.6
Habitat	Human wound secretions	Brackish lake	Small creek	Human blood	Freshwater lake

1, GX5518^T^; 2, *F. sanaruensis* JCM 31447^T^; 3, *F. multicolorata* 33A1-SZDP^T^; 4, "*P. ruber*" KCTC 72920^T^; 5, *S. aquatica* DSM 23856^T^

PE: phosphatidylethanolamine, PG: phosphatidylglycerol. Summed feature 3: C_16:1_ *ω*6c/C_16: 1_ *ω*7c. +, positive; −, negative; w, weakly positive; ND, not detected or we could not find related information in the reference paper. In the API ZYM and API 20NE strips, nitrate reduction, indole production, arginine dihydrolase, urease, lipase (C14), cystine arylamidase, *α*-mannosidase, *α*-chymotrypsin, *α*-galactosidase, *β*-galactosidase, *β*-glucuronidase, *α*-glucosidase, *β*-glucosidase, *α*-mannosidase, *β*-fucosidase, capric acid, adipic acid, malate, trisodium citrate and phenylacetic acid are all negative

*Data from Hahn et al. (2017), Maejima et al. (2021) and Pitt et al. (2020)

Susceptibility criteria were interpreted with reference to MIC breakpoint of other non-enterobacterium bacteria listed in CLSI M100. Antimicrobial susceptibility tests showed that isolate GX5518^T^ was sensitive to penicillins: penicillin (MIC = 2 µg/ml ≤ 16 µg/ml), ampicillin (MIC = 0.75 µg/ml ≤ 16 µg/ml), piperacillin (MIC = 1 µg/ml ≤ 16 µg/ml); carbapenems: meropenem (MIC = 0.008 µg/ml ≤ 4 µg/ml), imipenem (MIC = 0.25 µg/ml ≤ 4 µg/ml); fluoroquinolones: ciprofloxacin (MIC = 0.016 µg/ml ≤ 1 µg/ml), levofloxacin (MIC = 0.032 µg/ml ≤ 2 µg/ml); cephalosporins: ceftriaxone (MIC = 0.064 µg/ml ≤ 8 µg/ml), cefotaxime (MIC = 2 µg/ml ≤ 8 µg/ml); tetracyclines: tetracycline (MIC = 0.064 µg/ml ≤ 4 µg/ml), doxycycline (MIC = 0.064 µg/ml ≤ 4 µg/ml); chloramphenicol (MIC = 1 µg/ml ≤ 8 µg/ml), but was resistant to monoamide rings: aztreonam (MIC = 256 µg/ml ≥ 32 µg/ml); oxazolidinones: linezolid (MIC = 256 µg/ml ≥ 256 µg/ml); aminoglycosides: gentamicin (MIC = 16 µg/ml ≥ 16 µg/ml), vancomycin (MIC = 256 µg/ml ≥ 32 µg/ml).

### Chemotaxonomic characteristics

The major fatty acids (> 5% of total) in cells of isolate GX5518^T^ were iso-C_15:0_ (37.6%), C_16:0_ (13.9%), C_17:0_ (10.9%), C_16:1_ *ω*7*c*/C_16:1_ *ω*6*c* (summed feature 3, 6.7%) and C_17:1_ *ω*8*c* (6.0%) (Table S1). However, fatty acid compositions between isolate GX5518^T^ and *F. sanaruensis* JCM 31447^T^ were great difference, such as the amounts of iso-C_17:0_ and C_14:0_ in cells of isolate GX5518^T^ were higher than other relatives, and the amounts of anteiso-C_15: 0_ and summed feature 3 were lower than in *F. sanaruensis* JCM 31447^T^. Phosphatidylethanolamine (PE), phosphatidylglycerol (PG), and three unknown lipids (L1-3) (Fig. S3) were detected in isolate GX5518^T^. The predominant menaquinone was MK-8 (86.9%), which was similar to the other members in the family *Silvanigrellaceae.*

### Animal experiments and histopathological analysis

The results of animal virulence experiments showed that no mice died within the 7-day observation period of mice injected with isolate GX5518^T^ bacterial suspension intraperitoneally. Heart tissue was congested with focal inflammatory cell infiltration; inflammatory cell infiltration was seen between hepatic vessels and parenchyma, small bile duct hyperplasia, and a large number of inflammatory cells gathered and infiltrated next to the bile duct; lung tissue structure was acceptable, with regional alveolar collapse and fusion, inflammatory cell infiltration; A small amount of inflammatory cell infiltration was seen in the spleen and kidney. In summary, the main pathological changes are inflammatory reactions, with the heart, liver and lung being the most serious (Fig. S4). Based on animal virulence experiments, it was confirmed that GX5518^T^ has potential pathogenicity and virulence. Combined with the characteristics of the patient's case, the infection occurred after stone injury. Considering that such microorganisms mainly exist in the environment, they may be opportunistic pathogenic bacteria. Animal experiments suggested but could not completely rule out the possibility that the virulence of isolate GX5518^T^ was weakened or lost due to factors such as artificial passage or growth conditions of the medium.

### Phylogenetic and genome analysis

The 16S rRNA gene sequence of isolate GX5518^T^ (1469 bp) was submitted to the GenBank database with the accession number MT879459. Comparison of the 16S rRNA gene sequences showed that isolate GX5518^T^ shared the highest similarities with *F. sanaruensis* JCM 31447^T^ (99.73%) and *F. multicolorata* DSM 107810^T^ (98.49%)*.* Phylogenetic analyses based on the NJ, ML and ME methods clearly showed that isolate GX5518^T^ formed an independent branch in the genus *Fluviispira*, and was closely related to the cluster composed by *S. paludirubra* DSM 107809^T^ (95.15%), *S. aquatica* DSM 23856^T^ (94.75%) and “*P. ruber”* KCTC 72920^T^ (94.28%) in the family *Silvanigrellaceae* (Figs S5-S7).

**Fig. 2:**
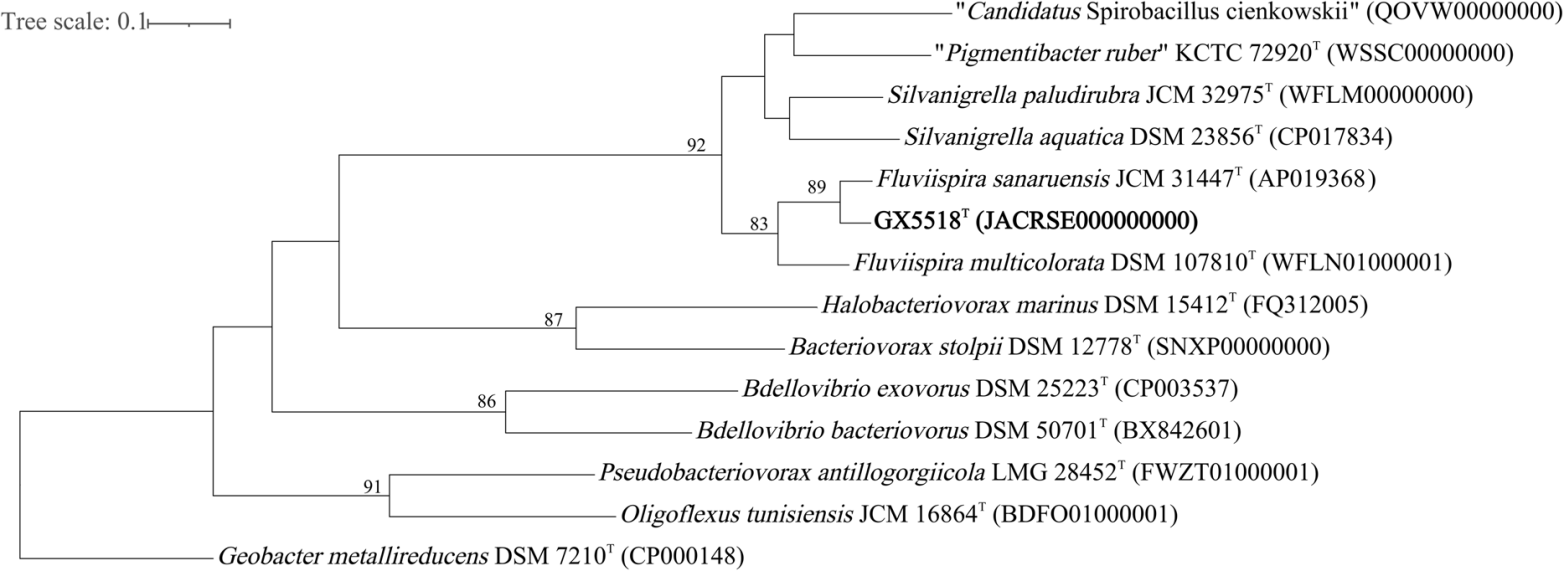
Phylogenomic tree based on 92 core genes contrasted by the software UBCG showing the relationship of isolate GX5518^T^ and closely related taxa. *Geobacter metallireducens* DSM 7210^T^ (CP000148) was used as the outgroup. Bootstrap values (> 50%) are shown at the branch nodes

The draft genome sequences of isolate GX5518^T^ have been deposited at DDBJ/ENA/GenBank under the accession JACRSE000000000, was 3,604,777 bp in length, which was composed of 9 contigs with an N50 of 580,334 bp, genomic DNA G + C content of 33.1% and coverage of 100×. Phylogenomic analyses with the software UBCG clearly showed that isolate GX5518^T^ belonged to the genus *Fluviispira*, and was most closely related to *F. sanaruensis* JCM 31447^T^ and *F. multicolorata* DSM 107810^T^ (Fig. 2), which were similar with the phylogenies based on the 16S rRNA gene sequence. The estimated ANIb, ANIm, AAI and dDDH values between isolate GX5518^T^ and its closely related type strains derived from phylogenomic analyses and 16S rRNA gene phylogenies were compared, with the ANIb ranging from 62.0 to 88.7% and the ANIm ranging from 83.18 to 92.13%, AAI values ranging from 44.46 to 91.87%, dDDH values were 12.9 to 63.7%, and compared to the closest species *F. sanaruensis* JCM31447^T^ were 88.67%, 89.5%, 91.87%, 63.7%, respectively, which were far lower than the threshold values for species delimitation (95–96% ANI, 95–96% AAI and 70% dDDH, respectively) (Meier-Kolthoff et al. 2013; Richter and Rosselló-Móra 2009; Chun et al. 2018). The results were shown in Table S2. In conclusion, although the 16S rRNA gene sequence similarities of the isolate GX5518^T^ were more than the species’ threshold limits (98.65%) (Kim et al. 2014), the genomic analysis results indicated that isolate GX5518^T^ could represented a novel genomic species in the genus *Fluviispira.*

Based on the genomes of isolate GX5518^T^ and its closely related species, the subsystem features were compared by using the RAST server and SEED viewer to indicate the presence or absence of gene-associated biological functions. A total of 3281 coding sequences (CDSs) with 40 RNAs were predicted for the novel strain, of which 498 (accounting for 16% of the total) were assigned into 365 features in the subsystem. Similar to those of closely related type strains, most of the assigned genes of the novel isolate were involved into the categories of amino acids and derivatives (121), protein metabolism (86), carbohydrates (86), cofactors/vitamins/prosthetic groups/pigments (68), fatty acids/lipids/isoprenoids (58). The detailed comparison of Subsystem features distribution between isolate GX5518^T^ and its related strains is provided in Table S3. Interestingly, genes involved in resistance to antibiotics and toxic compounds in isolate GX5518^T^ including copper homeostasis (7), cobalt-zinc-cadmium resistance (1), resistance to fluoroquinolones (2), and Zinc resistance(1). The heavy metals are hazardous and contaminate the environment and adversely affects the quality of the soil, isolate GX5518^T^ may have a role in resisting heavy metal toxicity and increasing heavy metal removal efficiency.

**Fig. 3:**
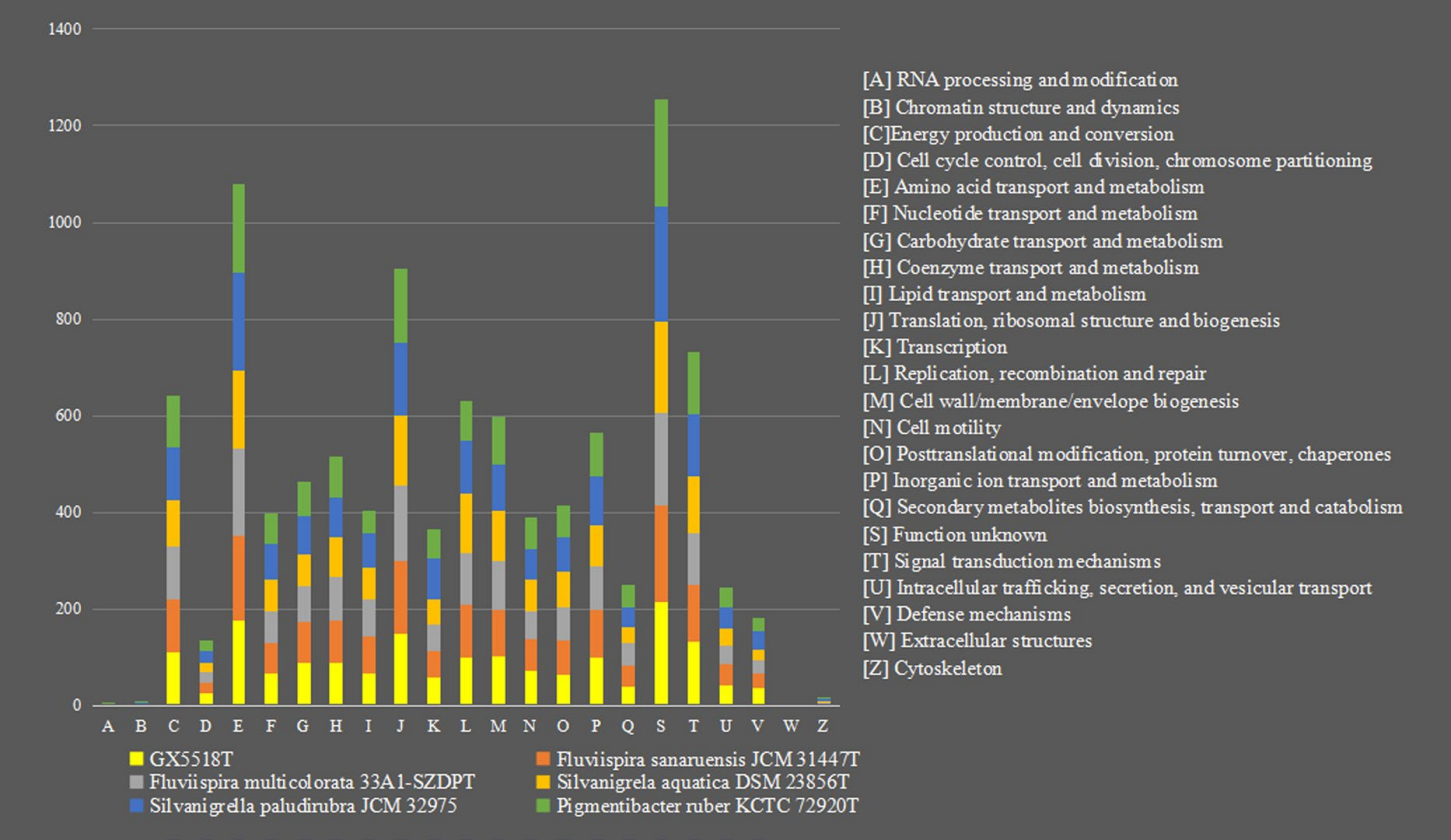
Comparison of genes based on the 23 general eggNOG functional categories of isolate GX5518^T^ with its phylogenetically related species of the family *Silvanigrellaceae*

Annotation of the Cluster of Orthologous Groups [(COGs) (of proteins)] for the isolate GX5518^T^ found that a total of 1724 genes were assigned to 23 functional categories. Among the obtained functional groups, the cluster for [E] (amino acid transport and metabolism; 175), [C] (Energy production and conversion; 111), [J] (Translation, ribosomal structure and biogenesis; 149), [M] (Cell wall/membrane/envelope biogenesis; 101), [T] (Signal transduction mechanisms; 131), [P] (Inorganic ion transport and metabolism; 99), [L] (Replication, recombination and repair; 98), [G] (Carbohydrate transport and metabolism; 88), [H] (Coenzyme transport and metabolism; 88) were the most highly represented categories. Compared with the *F. sanaruensis* JCM 31447^T^, more numbers of the Signal transduction mechanisms in the isolate GX5518^T^. The comparison of the class of protein function and its number between isolate GX5518^T^ and its related strains is provided in Fig. 3.

In addition, the antibiotic resistance genes and virulence genes of isolate GX5518^T^ were predicted. For the CARD database (version 3.2.2), RGI 6.0.1 software, including Perfect, Strict and Loose algorithms, was used as the screening criteria. Five AMR Gene Family (elfamycin resistant EF-Tu, isoniazid resistant katG, resistance-nodulation-cell division (RND) antibiotic efflux pump, rifamycin-resistant beta-subunit of RNA polymerase (rpoB), and antibiotic resistant fusE) have been found in the genomes of isolate GX5518^T^. EF-Tu is a elongation factor tu gtp-binding domain protein 2, which responsible for the elongation factor of peptide chains during protein synthesis and plays important role in ribosome translation proteins. Expression of the EF-Tu variant is conferred in the resistance to elfamycin. Catalase and peroxidase encoded by *katG* gene plays an important role in the oxidative metabolism process of bacteria, and mutations in the *katG* gene are more common cause of isoniazid resistance. RND antibiotic efflux pump involves three genes: *MuxB*, *mdtC*, and *MexW*, which is conferred in the resistance to macrolide antibiotic, monobactam, tetracycline antibiotic, aminocoumarin antibiotic, fluoroquinolone antibiotic and phenicol antibiotic. *RpoB* mutants and *fusE* mutants confer resistance to conferring resistance to rifampicin and fusidane antibiotic, respectively.

Based on the VFDB database, 8 virulence-related genes, *htpB*, *cheY*, *hemB*, *clpB*, *eno*, *cps2L*, *fliQ*, *katA* were detected in the genome of isolate GX5518^T^. Here, we also compared the virulence-related gene annotation results with “*P. ruber”* KCTC 72920^T^, which is currently the only pathogenic bacterium that causes human infections in the family *Silvanigrellaceae.* Compared to “*P. ruber”* KCTC 72920^T^, we observed the three genes, *cps2L*, *fliQ*, and *katA* are unique in isolate GX5518^T^. *cps2L*, encoding glycosyltransferases involved in capsular polysaccharide (CPS) synthesis, which can resistant to complement deposition and masks cell wall-associated complement from being recognized by the complement receptors on phagocytes. Furthermore, the CPS is an essential virulence factor for isolate GX5518^T^ to infect the host. The flagellar biosynthesis protein FliQ encoded by the *fliQ* gene is required for the assembly of the rivet at the earliest stage of flagellar biosynthesis. The catalase encoded by the *katA* gene participates in the decomposition of hydrogen oxidation and protects cells from the toxicity of hydrogen peroxide. Stable and highly active KatA plays an important role in maintaining its own oxygen metabolism balance, enabling strains to escape oxidative damage from neutrophils and macrophages and survive in adverse environment.

## Taxonomic conclusion

Similar morphological characterization and the high 16S rRNA gene similarities suggested that isolate GX5518^T^ was closely related to the genus *Fluviispira*. While, the dDDH values, ANIb values, ANIm values, AAI values, and chemotaxonomic characteristics between the isolate GX5518^T^ and its closely related strains have proved that it represented a novel species of the genus *Fluviispira.* In conclusion, based on genotypic, phenotypic, biochemical characteristics and phylogenetic analysis, it is proposed that isolate GX5518^T^ (= CGMCC 1.18685^T^ = KCTC 82149^T^) represented a novel species of the genus *Fluviispira*, for which the name *Fluviispira vulneris* sp. nov. is proposed.

### Description of *Fluviispira vulneris* sp. nov.

*Fluviispira vulneris* (vul’ne.ris. L. gen. neut. n. *vulneris*, of a wound in which the organism was isolated).

Cells are aerobic, Gram-negative, non-motile and pleomorphic. The colony is round, smooth, convex, with red-pigmentation. Growth occurs on Columbia blood agar, chocolate agar, R2A agar, Blood-R2A agar, BCYEα agar, Blood-TSA agar and Blood-MH agar. Growth occurs at 10–37 °C (optimum, 28–32 °C), pH 6.0–8.0 (optimum, pH 7.0) and the salt tolerance up to 1.5% (w/v). In the API ZYM and API 20NE strips, it is positive for alkaline phosphatase, leucine arylamidase and acid phosphatase, and weakly positive for esculin, esterase (C4), esterase lipase (C8), valine arylamidase, naphthol-AS-BI-phosphohydrolase and *N*-acetyl-glucoslaminase. Sensitive to penicillin, ampicillin, piperacillin, meropenem, imipenem, ciprofloxacin, levofloxacin, ceftriaxone, cefotaxime, tetracycline, doxycycline, chloramphenicol, but resistant to aztreonam, linezoli, gentamicin, and vancomycin. The predominant fatty acids (> 5%) in isolate GX5518^T^ cells are iso-C_15:0_, C_16:0_, C_17:0_, C_17:1_ ω8c and C_16:1_ ω7c/C_16:1_ ω6c. The major menaquinone is MK-8. The polar lipids are phosphatidylethanolamine, phosphatidylglycerol and three unknown lipids (L1-3).

The type strain is GX5518^T^ (= CGMCC 1.18685^T^ = KCTC 82149^T^), which was isolated from human wound secretions in Guangxi, PR China. The genomic DNA G + C content is about 33.1 mol%. The 16S rRNA gene sequences of the type strain GX5518^T^ is available under the GenBank accession numbers MT879459. The GenBank accession number for the draft genome of the type isolate GX5518^T^ is JACRSE000000000 at GenBank/EMBL/DDBJ/PIR.

### Supplementary Information

Below is the link to the electronic supplementary material.

Supplementary file1 (DOCX 24 KB)

Supplementary file2 (PNG 7877 KB)

Supplementary file3 (JPG 2231 KB)

Supplementary file4 (TIF 5326 KB)

Supplementary file5 (PNG 1287 KB)

Supplementary file6 (PNG 1210 KB)

Supplementary file7 (PNG 1378 KB)

Supplementary file8 (PNG 5133 KB)

Supplementary file9 (PNG 12639 KB)

Supplementary file10 (PNG 6520 KB)

Supplementary file11 (PNG 7499 KB)

Supplementary file12 (PNG 17099 KB)

Supplementary file13 (PNG 12843 KB)

Supplementary file14 (PNG 8248 KB)

Supplementary file15 (PNG 2459 KB)

Supplementary file16 (PNG 2485 KB)

Supplementary file17 (PNG 2484 KB)

## Abbreviations

AAI: Average amino acid identity
ANI: Average nucleotide identity
dDDH: Digital DNA–DNA hybridization
G + C: Guanine-plus-cytosine
MALDI-TOF MS: Matrix-assisted laser desorption ionization-time of flight mass spectrometry
Blood-R2A agar: R2A agar supplemented with 5% sheep red blood
BCYEα: Buffered charcoal yeast extract medium supplemented with a-ketoglutaric acid
BHI: Brain heart infusion
MH: Mueller–Hinton
MIC: Minimum inhibitory concentration
MEGA: Molecular evolutionary genetics analysis
NJ: Neighbor-joining
ML: Maximum-likelihood
CLSI: The clinical and laboratory standards institute
HPLC: High performance liquid chromatography
