# Supplementary material for: Fluviispira vulneris sp. nov., isolated from human wound secretions
Source: Antonie Van Leeuwenhoek. 2023 Sep 29;116(12):1305–16. doi: 10.1007/s10482-023-01883-4 (PMC10645651; doi:10.1007/s10482-023-01883-4)
Supplement: Supplementary file 1 — Supplementary file1 (DOCX 24 KB) [file 10482_2023_1883_MOESM1_ESM.docx]

***Fluviispira vulneris* sp. nov., isolated from human wound secretions**

**Peijuan Tang ^.^ Na Peng ^.^ Pengwen Ouyang ^.^ Sheng Long ^.^ Zhenhua Wei ^.^ Xingchun Chen ^.^ Pinghua Qu ^.^ Liangyi Xie**

P.-J. Tang **^.^** N. Peng **^.^** P.-W. Ouyang **^.^** S.-Long **^.^** L.-Y. **^.^** Xie (🖂)

Department of Clinical Laboratory, Hunan Province People’s Hospital, The First Affiliated Hospital of Hunan Normal University, Changsha 410005, People’s Republic of China.

Z.-H. Wei

Mashan County People’s Hospital, Guangxi 530600, People’s Republic of China.

X.-C. Chen

Guangxi Province People’s Hospital, Guangxi 530000, People’s Republic of China.

P.-H. Qu (🖂)

Department of Clinical Laboratory, The Second Clinical College of Guangzhou University of Chinese Medicine, Guangdong Provincial Hospital of Traditional Chinese Medicine, Guangzhou 510006, People’s Republic of China.

🖂 Liangyi Xie

E-mail: [lyxie78@hunnu.edu.cn](mailto:lyxie78@hunnu.edu.cn)

🖂 Pinghua Qu

E-mail: Ping-Hua Qu, [ququtdr@163.com](mailto:ququtdr@163.com;)

**Table S1** Cellular fatty acid compositions of (ratio %) isolate GX5518^T^ and its related species

| Fatty acids (%) | 1 | 2 | 3 | 4 |
| --- | --- | --- | --- | --- |
| Saturated straight chain: | | | | |
| C_14:0_ | 4.9 | 2.5 | 0.9 | 1.2 |
| C_16:0_ | **13.9** | **14.1** | 2.3 | **14.8** |
| C_17:0_ | **10.9** | 2.4 | 5.8 | 6.0 |
| Unsaturated straight chain: | | | | |
| C_15:1_ *ω*6*c* | 0.6 | tr | 3.1 | tr |
| C_17: 1_ *ω*8*c* | 6.0 | 2.0 | **12.0** | 2.0 |
| C_18:1_ *ω*9*c* | 1.5 | 2.0 | - | 2.4 |
| Saturated branched-chain: | | | | |
| iso-C_14:0_ | 1.1 | 1.1 | 11.1 | tr |
| iso-C_15:0_ | **37.6** | **37.9** | **20.8** | **47.7** |
| anteiso-C_15: 0_ | 3.5 | 9.3 | 9.3 | 2.0 |
| iso-C_16: 0_ | tr | 0.6 | 9.8 | tr |
| iso-C_17: 0_ | **1.7** | 6.2 | 1.1 | 5.2 |
| anteiso-C_17: 0_ | tr | 0.7 | tr | 0.7 |
| Hydroxy acids: | | | | |
| C_10:0_-3-OH | tr | tr | 2.4 | - |
| C_11:0_-3-OH | 1.1 | tr | - | - |
| iso-C_11:0_-3-OH | tr | tr | - | - |
| C_12: 0_−3-OH | tr | tr | 2.1 | 0.8 |
| C_16: 0_−3-OH | 1.4 | 1.6 | tr | 0.8 |
| C_17 :0_−3-OH | 2.5 | 1.0 | 1.9 | tr |
| iso-C_17: 0_−3-OH | 0.6 | 1.6 | tr | 1.8 |
| *Summed feature: |  |  |  |  |
| 1 | tr | tr | - | 0.8 |
| 3 | 6.7 | **10.6** | 4.5 | 5.8 |
| 5 | tr | 1.3 | - | 1.0 |

1, GX5518^T^; 2, *F.*  *sanaruensis* JCM 31447^T^. 3, *F.*  *multicolorata* JCM 32978^T^ (Pitt et al. 2020); 4, “*P.*  *ruber”* KCTC 72920^T^*.* *Summed feature 1: iso-C_15:1_ iH/C_13:0_-3OH, summed feature 3: C_16 :1_ *ω*6*c*/C_16 : 1_ *ω*7*c*. summed feature 5: C_18:0_ ante/C_18:2_ *ω*6,9*c*；summed feature 8: C_18:1_ *ω*7*c*. “tr” and “−” indicate trace (< 0.5 %) and not detected, respectively. Only percentages higher than 0.5% are listed. Values are percentages of the total fatty acids. Fatty acids with more than 10% are in bold.

**Table S2.** ANI and AAI values between genomes of isolate GX5518^T^ and that of related species of the family *Silvanigrellaceae*

| Strains (accession number) | Size (×10^6^) | G+C (%) | ANIb (%) | ANIm (%) | AAI (%) | DDH  (%) |
| --- | --- | --- | --- | --- | --- | --- |
| *Silvanigrella* *aquatica* DSM23856^T^ (CP017834) | 3.42 | 32.9 | 70.34 | 83.31 | 67.21 | 14.8 |
| *Silvanigrella* *paludirubra* JCM 32975^T^ (WFLM00000000) | 3.94 | 29.3 | 70.84 | 83.24 | 66.83 | 15.1 |
| *Fluviispira* *multicolorata* JCM 32978^T^ (WFLN01000001) | 3.39 | 32.2 | 77.35 | 83.43 | 79.2 | 12.9 |
| *Fluviispira* *sanaruensis* JCM 31447^T^ (AP019368) | 3.67 | 33.6 | 88.67 | 89.5 | 91.87 | 63.7 |
| *“Pigmentibacter* *ruber”* KCTC 72920^T^ (WSSC00000000) | 3.64 | 29.6 | 69.52 | 83.18 | 64.64 | 14.1 |
| *“Spirobacillus* *cienkowskii”* (QOVW00000000) | 2.74 | 32.1 | 69.28 | 83.6 | 64.85 | 13.9 |
| *Bacteriovorax* *stolpii* DSM 12778^T^ (NZ_CP025704) | 3.81 | 40.9 | 62.58 | * | 44.63 | 12.9 |
| *Bdellovibrio* *bacteriovorus* DSM 50701^T^ (NC_005363) | 3.78 | 50.6 | 62.52 | 0 | 45.01 | 12.9 |
| *Bdellovibrio* *exovorus* JSS^T^ (NC_020813) | 2.66 | 41.9 | 62.54 | 0 | 45.62 | 12.9 |
| *Halobacteriovorax* *marinus* DSM 15412^T^ (NC_016620) | 3.44 | 36.7 | 62.17 | 0 | 44.46 | 12.9 |
| *Oligoflexus* *tunisiensis* JCM 16864^T^ (BDFO01000001) | 7.57 | 54.3 | 62.73 | 92.13 | 46.01 | 12.9 |
| *Pseudobacteriovorax* *antillogorgiicola* LMG 28452^T^ (FWZT01000001) | 7.28 | 45.9 | 62.94 | 0 | 46.08 | 12.9 |
| *Geobacter* *metallireducens* DSM 7210^T^ (NC_007517) | 4.01 | 59.5 | 62.00 | 0 | 44.97 | 12.9 |

**Table S3** Subsystem features distribution in the RAST system of isolate GX5518^T^ and closely related type strains.

Isolates: 1, GX5518^T^; 2, *F.* *sanaruensis* JCM 31447^T^; 3, *F.* *multicolorata* JCM 32978^T^. The numbers indicated the CDS numbers in the RAST subsystem.

| **Subsystem** **feature** | **1** | **2** | **3** |
| --- | --- | --- | --- |
| **Amino acids and derivatives** | 121 | 125 | 140 |
| Lysine/threonine/methionine/cysteine | 39 | 41 | 44 |
| Alanine/serine/glycine | 27 | 29 | 31 |
| Protein Metabolism | 86 | 87 | 90 |
| **Carbohydrates** | 86 | 91 | 96 |
| Central carbohydrate metabolism | 46 | 50 | 50 |
| Cofactors/Vitamins/Prosthetic Groups/Pigments | 68 | 71 | 64 |
| **Fatty Acids/Lipids/Isoprenoids** | 58 | 59 | 56 |
| Isoprenoids | 17 | 17 | 25 |
| Fatty acids | 16 | 14 | 19 |
| Phospholipids | 13 | 16 | 0 |
| DNA Metabolism | 49 | 47 | 49 |
| Nucleosides and Nucleotides | 40 | 42 | 43 |
| RNA Metabolism | 33 | 30 | 31 |
| Stress Response | 28 | 28 | 24 |
| Respiration | 25 | 29 | 27 |
| Cell Wall and Capsule | 23 | 28 | 26 |
| Virulence/Disease/Defense | 21 | 29 | 26 |
| Phosphorus Metabolism | 11 | 11 | 8 |
| Membrane Transport | 9 | 7 | 6 |
| Regulation and Cell signaling | 8 | 7 | 7 |
| Metabolism of Aromatic Compounds | 7 | 7 | 7 |
| Potassium metabolism | 6 | 6 | 6 |
| Phages/Prophages/Transposable elements/Plasmids | 5 | 3 | 1 |
| Sulfur Metabolism | 5 | 7 | 6 |
| Nitrogen Metabolism | 4 | 4 | 3 |
| Miscellaneous | 3 | 3 | 3 |
| Cell Division and Cell Cycle | 3 | 3 | 3 |
| Dormancy and Sporulation | 1 | 1 | 2 |

**Fig S1** Colonial morphology of isolate GX5518^T^ grown on BA agar for 3 days at 35℃.

**Fig S2**. Gram stained cells of isolate GX5518^T^ grown on BA agar for 3 days at 35℃.

**Fig S3** Two-dimensional thin layer chromatogram of the polar lipids of isolate GX5518^T^. Abbreviation: L1-3, unidentified lipid; PG, diphosphatidylglycerol; PE, phosphatidylethanolamine.

**Fig. S4.** Pathological changes in mice 5 days after infection with isolate GX5518^T.^ A: Heart; B: Liver; C: Spleen; D: Lung; E: Kidney. 1 is negative control, 2 is isolate GX5518^T^.

**Fig. S5.** Neighbour-joining tree based on 16S rRNA gene sequences, showing the relationship between isolate GX5518^T^ and its closely related species. Bar, 0.01 substitutions per nucleotide position. Numbers at nodes are bootstrap percentages >50 % (based on 1000 resamplings). *Geobacter metallireducens* DSM 7210^T^ (NR_025895) was used as the outgroup.

**Fig. S6.** Maximum-likelihood tree based on 16S rRNA gene sequences, showing the relationship between isolate GX5518^T^ and its closely related species. Bar, 0.01 substitutions per nucleotide position. Numbers at nodes are bootstrap percentages >50 % (based on 1000 resamplings). *Geobacter metallireducens* DSM 7210^T^ (NR_025895) was used as the outgroup.

**Fig. S7.** Maximum-parsimony tree based on 16S rRNA gene sequences, showing the relationship between isolate GX5518^T^ and its closely related species. Bar, 0.01 substitutions per nucleotide position. Numbers at nodes are bootstrap percentages >50 % (based on 1000 resamplings). *Geobacter metallireducens* DSM 7210^T^ (NR_025895) was used as the outgroup.
