# Supplementary figures and images for: Fluviispira vulneris sp. nov., isolated from human wound secretions
Source: Antonie Van Leeuwenhoek. 2023 Sep 29;116(12):1305–16. doi: 10.1007/s10482-023-01883-4 (PMC10645651; doi:10.1007/s10482-023-01883-4)

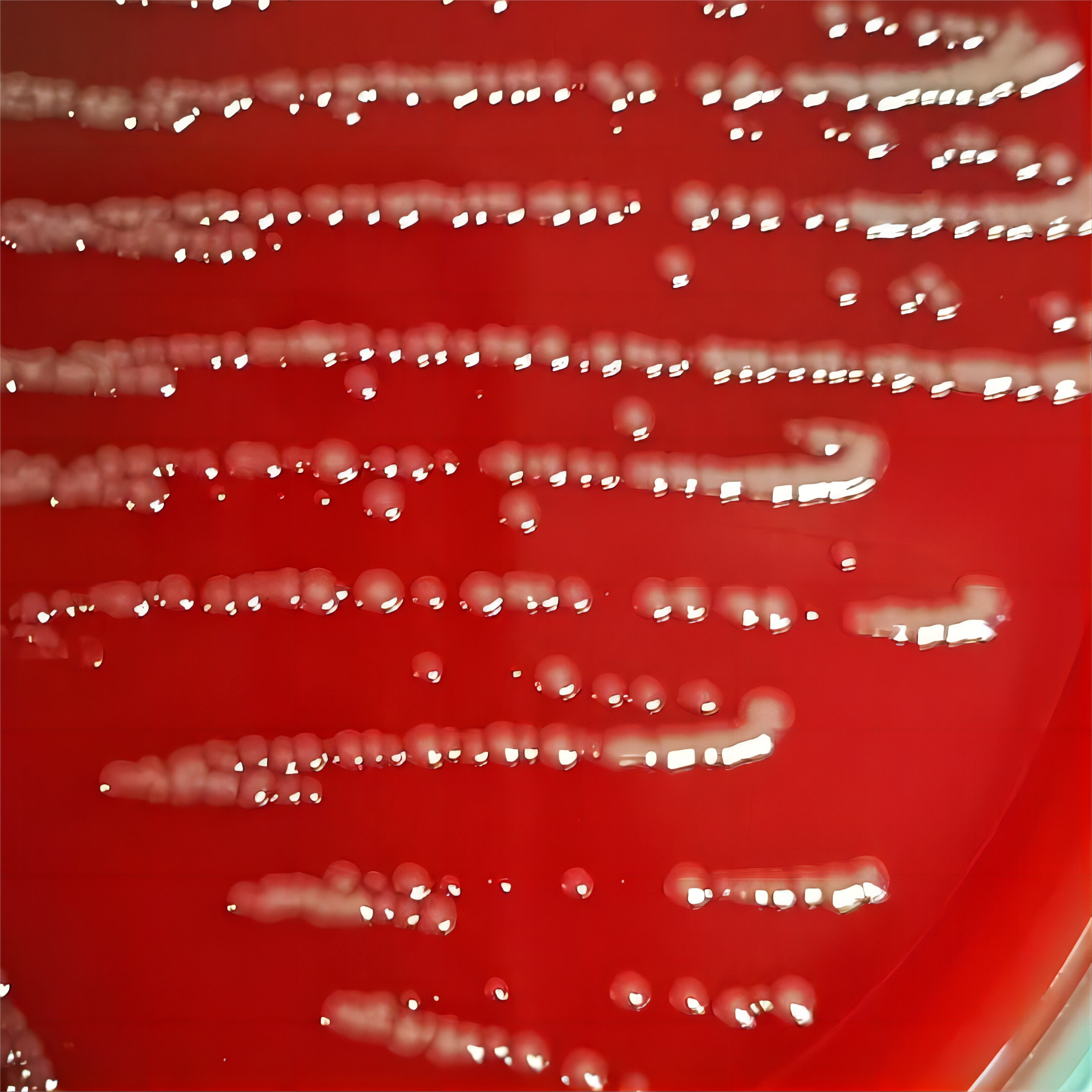

Supplement: Supplementary file 2 — Supplementary file2 (PNG 7877 KB) [file 10482_2023_1883_MOESM2_ESM.png]

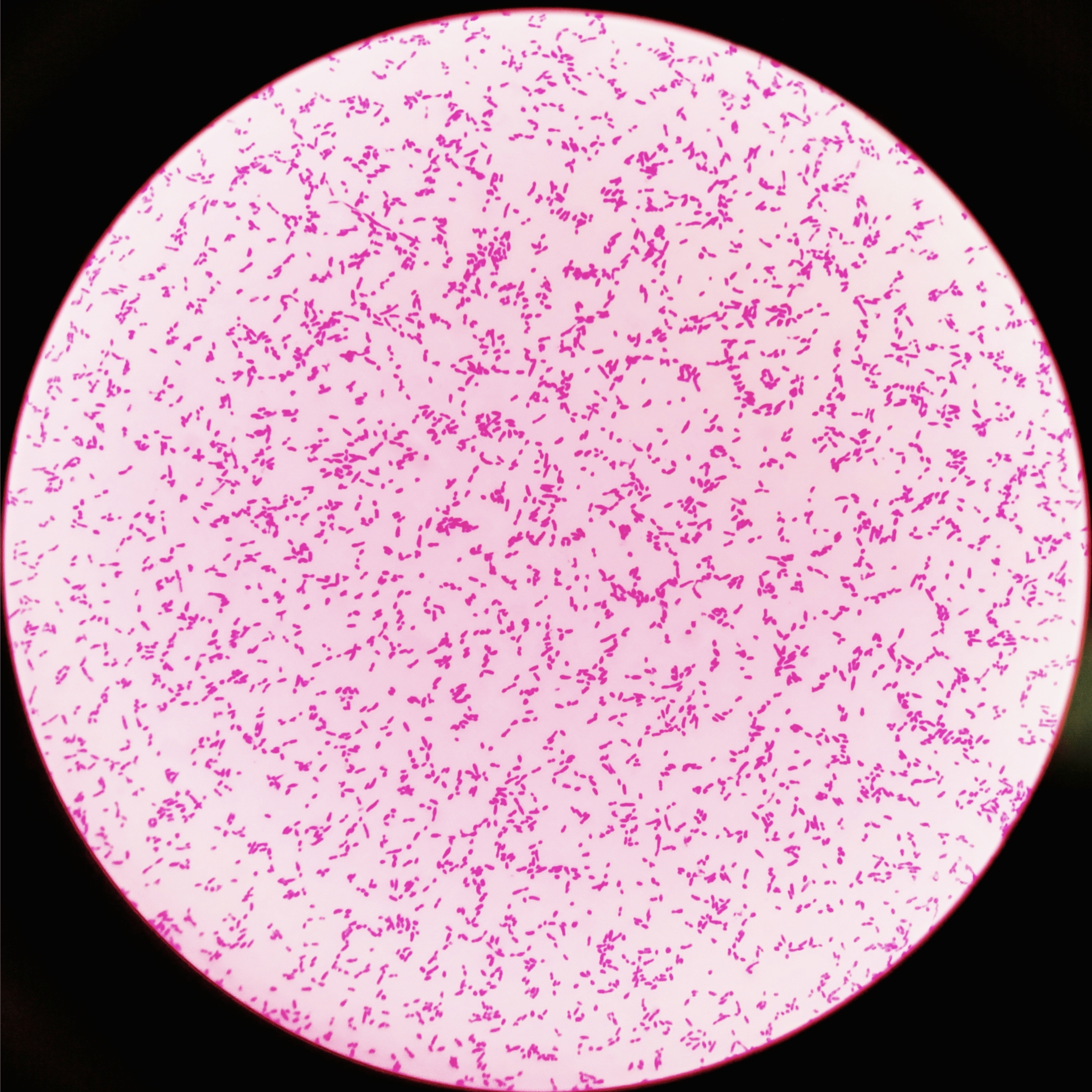

Supplement: Supplementary file 3 — Supplementary file3 (JPG 2231 KB) [file 10482_2023_1883_MOESM3_ESM.jpg]

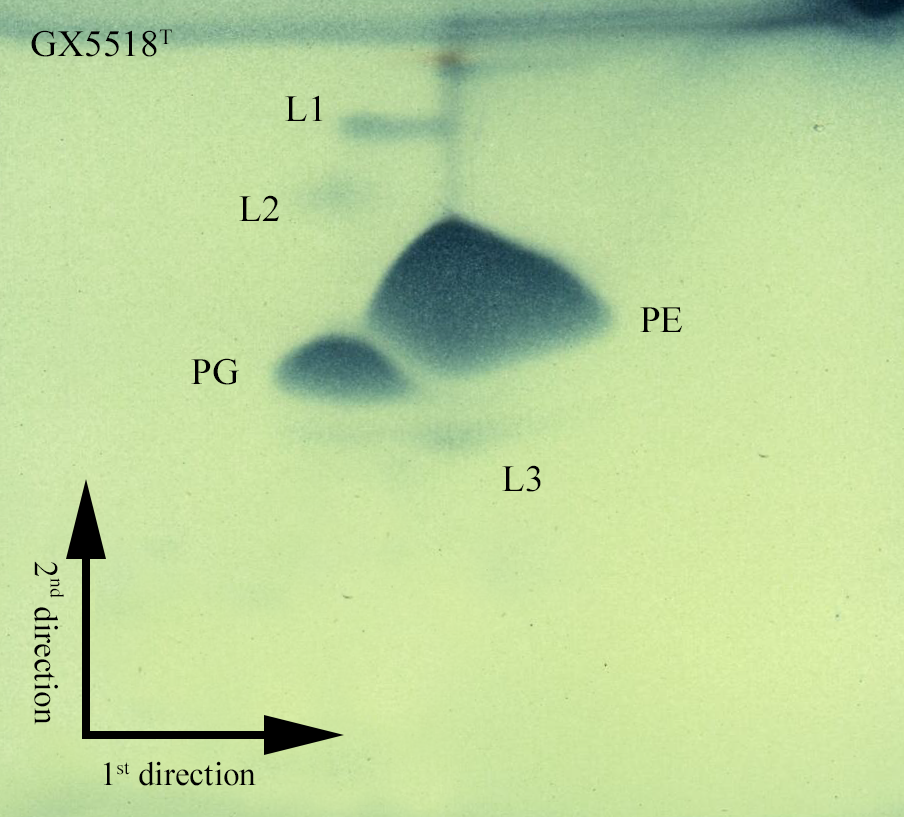

Supplement: Supplementary file 4 — Supplementary file4 (TIF 5326 KB) [file 10482_2023_1883_MOESM4_ESM.tif]

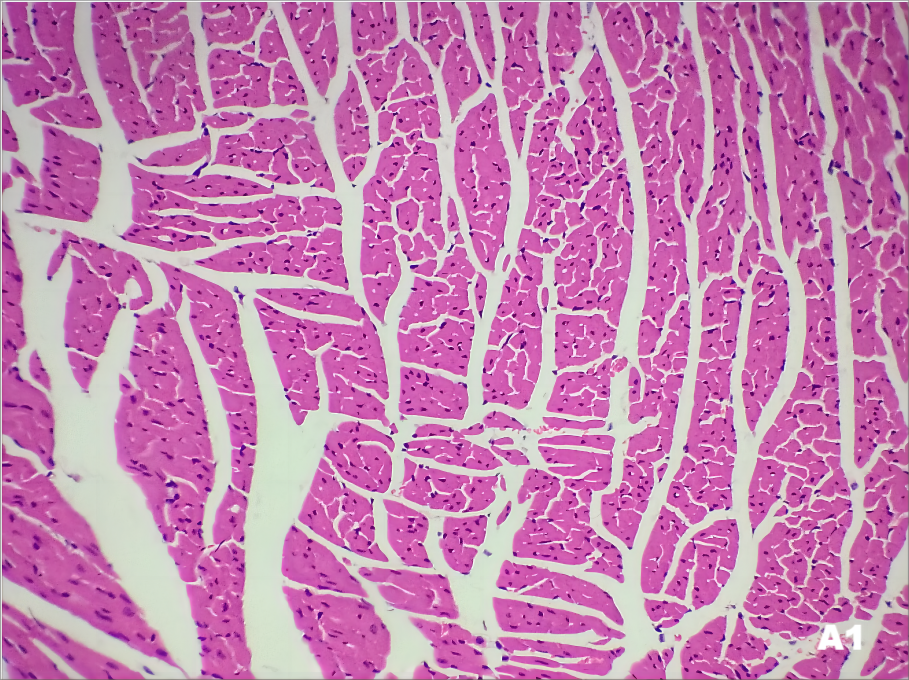

Supplement: Supplementary file 5 — Supplementary file5 (PNG 1287 KB) [file 10482_2023_1883_MOESM5_ESM.png]

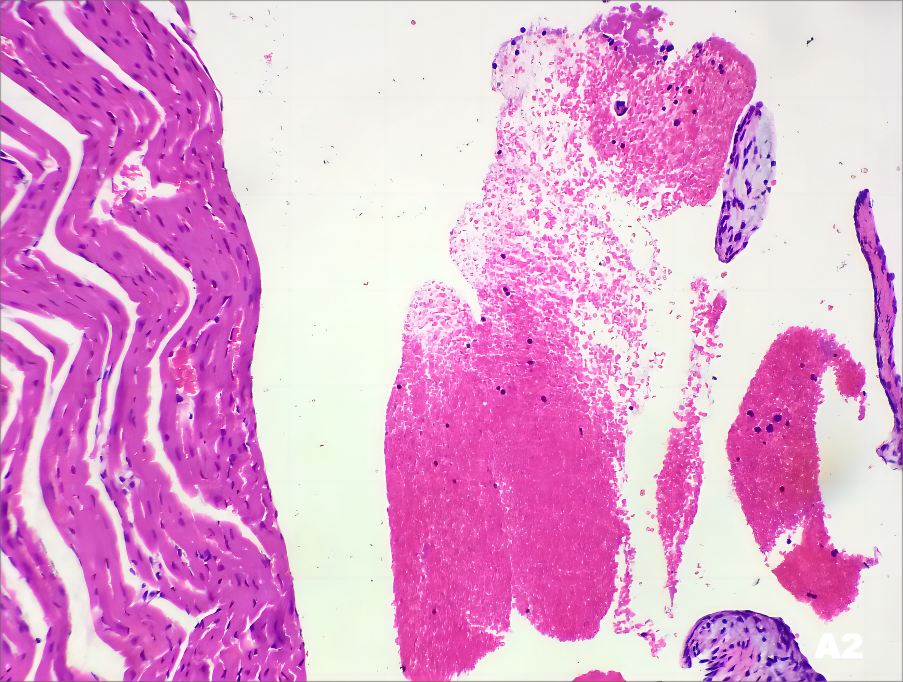

Supplement: Supplementary file 6 — Supplementary file6 (PNG 1210 KB) [file 10482_2023_1883_MOESM6_ESM.png]

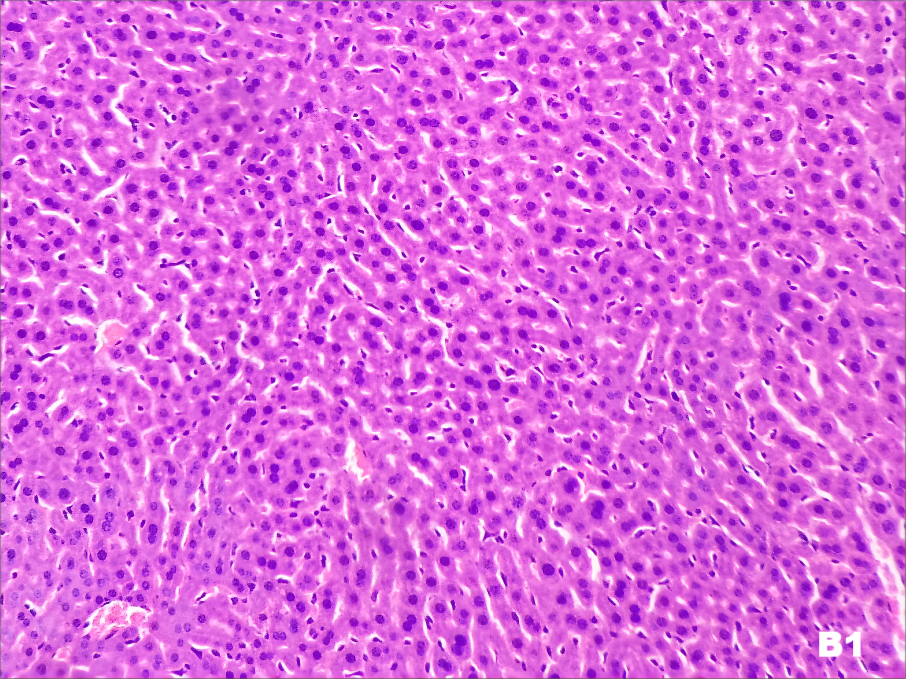

Supplement: Supplementary file 7 — Supplementary file7 (PNG 1378 KB) [file 10482_2023_1883_MOESM7_ESM.png]

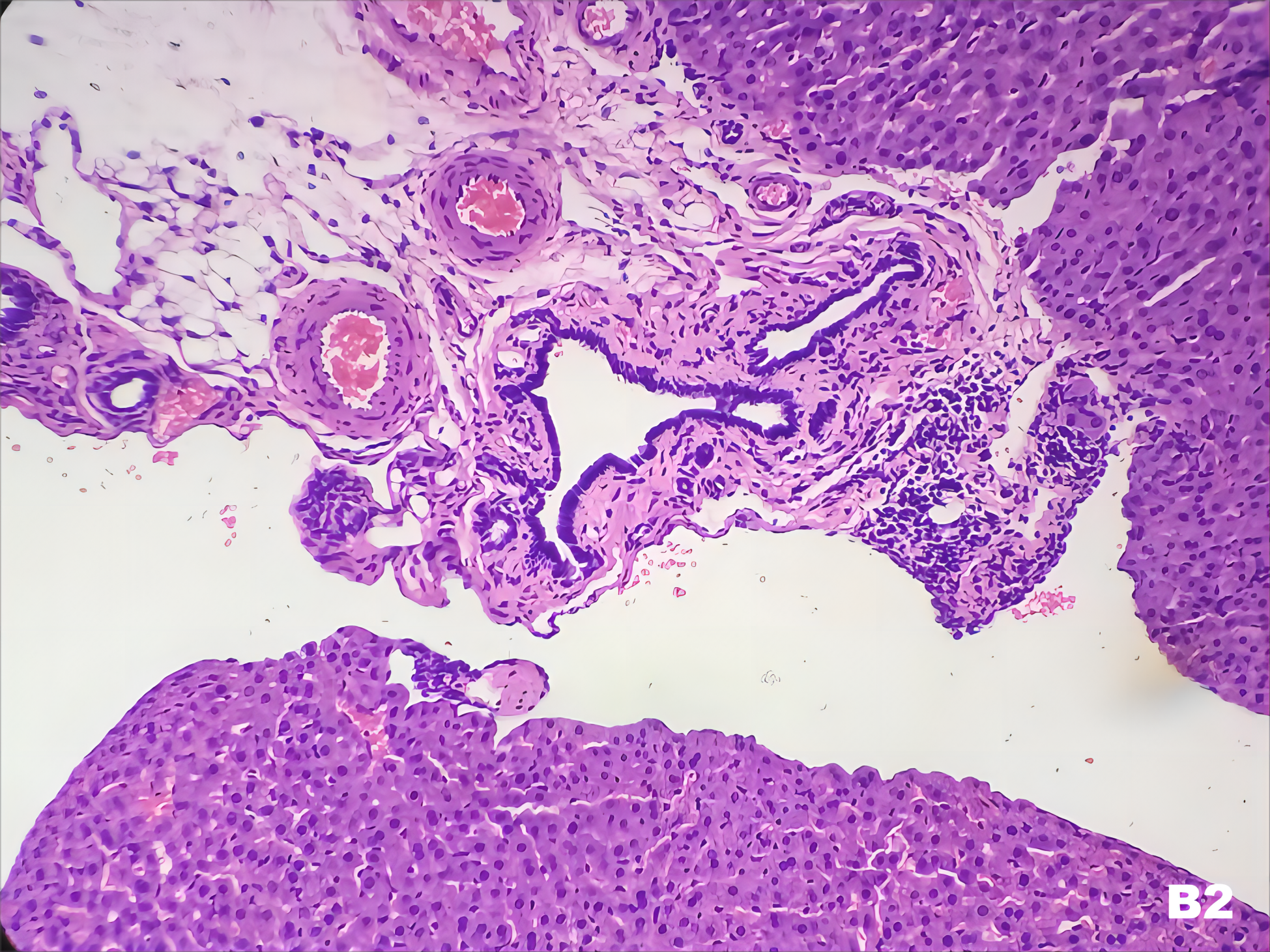

Supplement: Supplementary file 8 — Supplementary file8 (PNG 5133 KB) [file 10482_2023_1883_MOESM8_ESM.png]

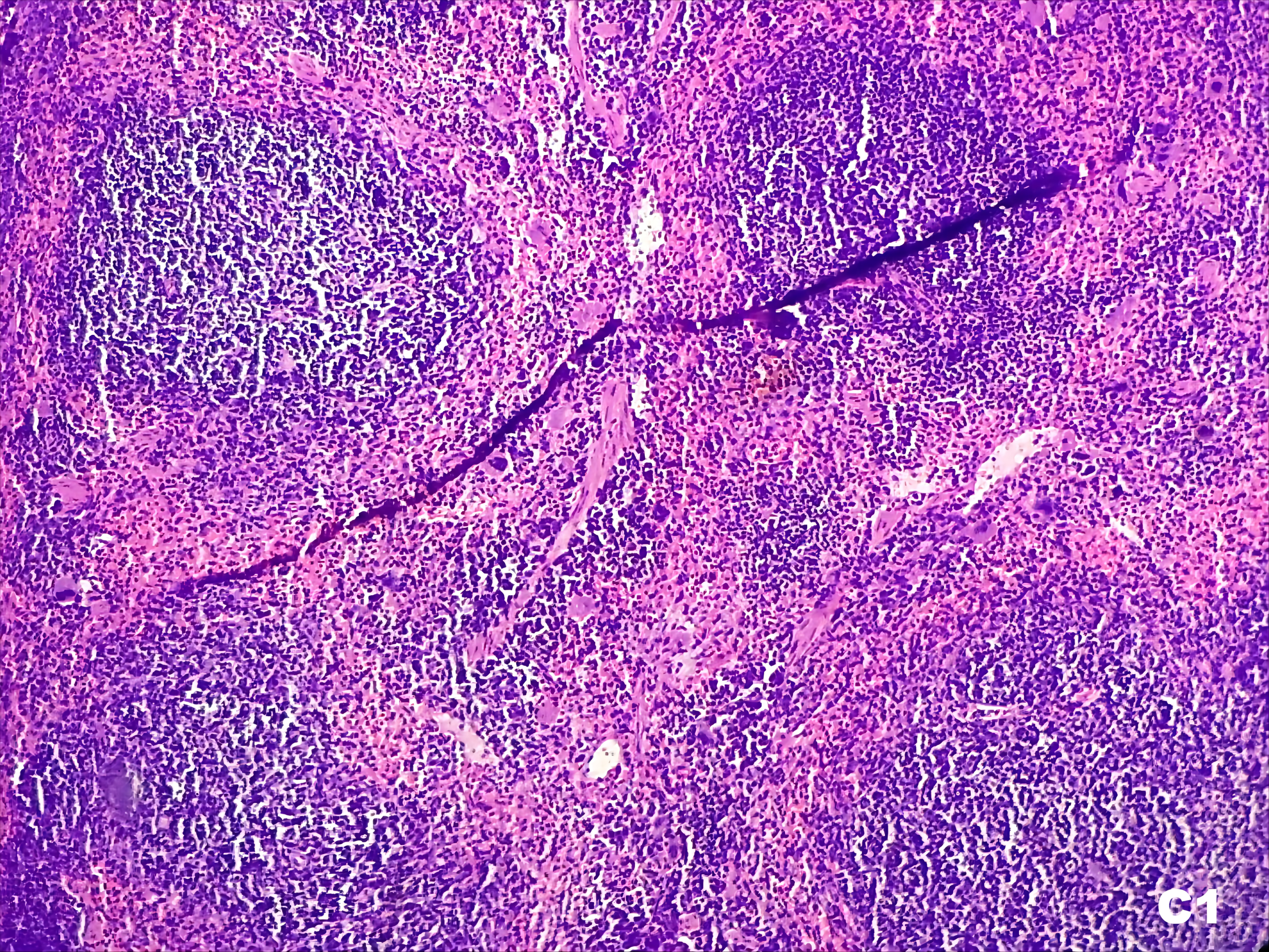

Supplement: Supplementary file 9 — Supplementary file9 (PNG 12639 KB) [file 10482_2023_1883_MOESM9_ESM.png]

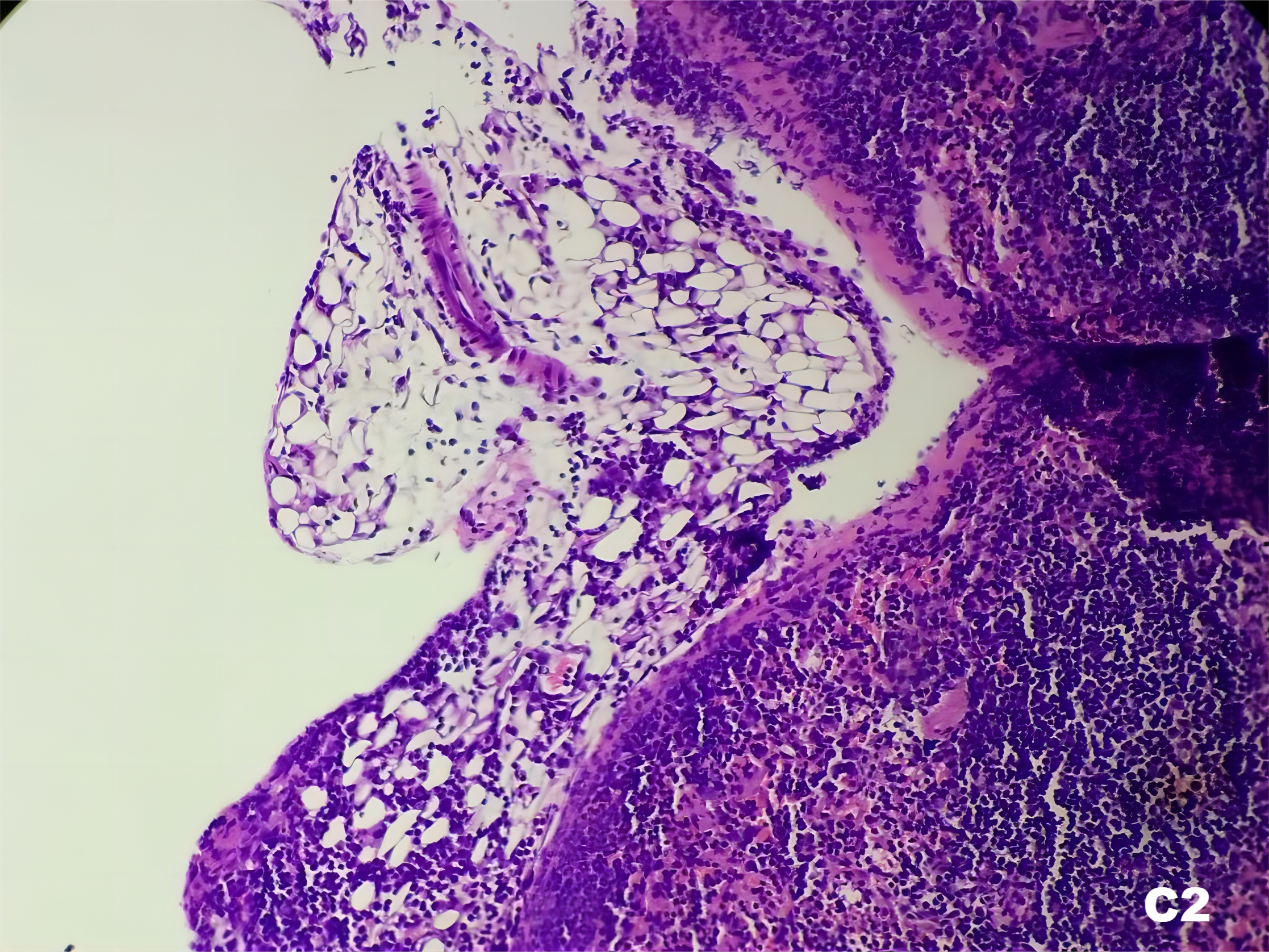

Supplement: Supplementary file 10 — Supplementary file10 (PNG 6520 KB) [file 10482_2023_1883_MOESM10_ESM.png]

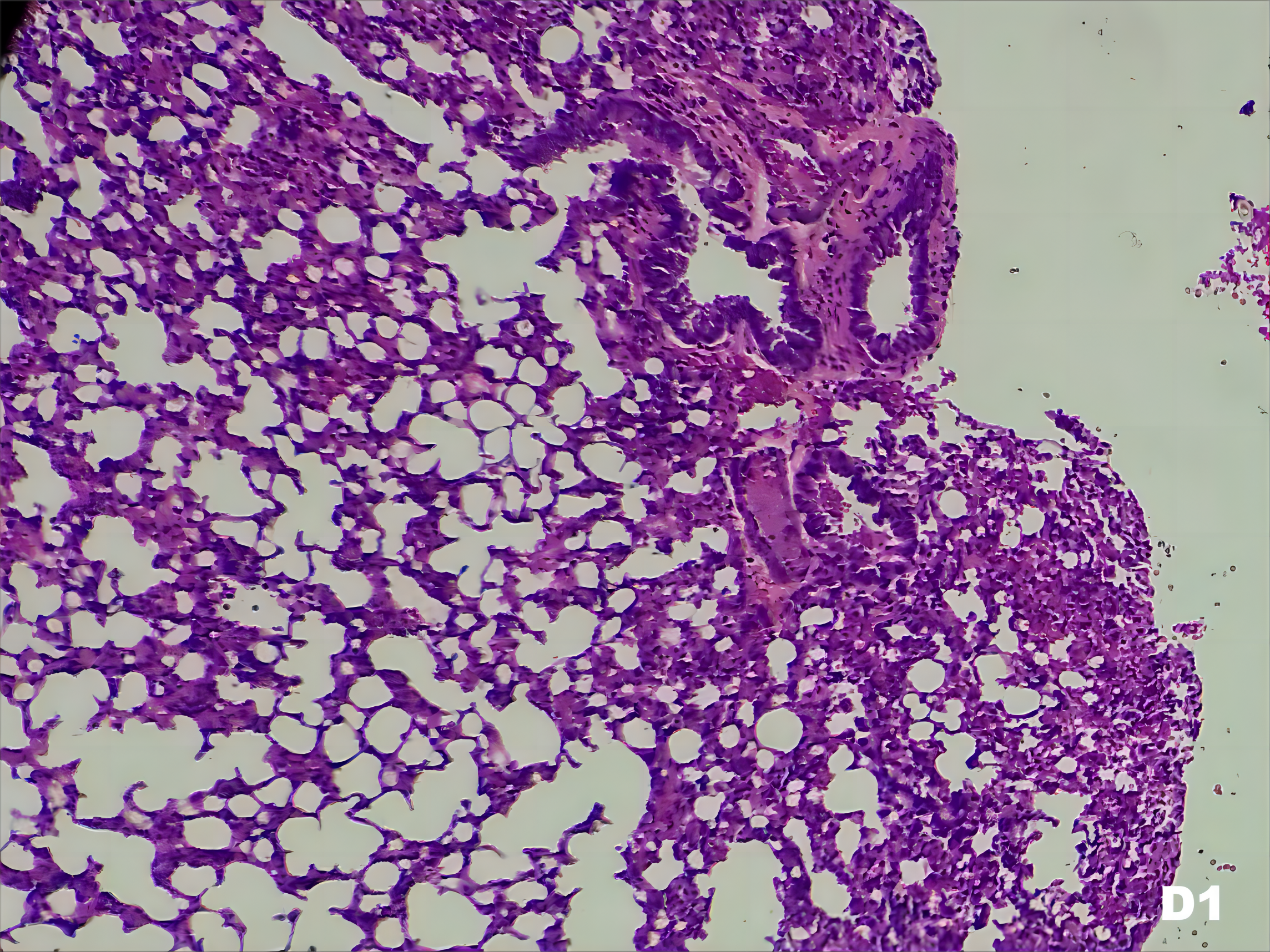

Supplement: Supplementary file 11 — Supplementary file11 (PNG 7499 KB) [file 10482_2023_1883_MOESM11_ESM.png]

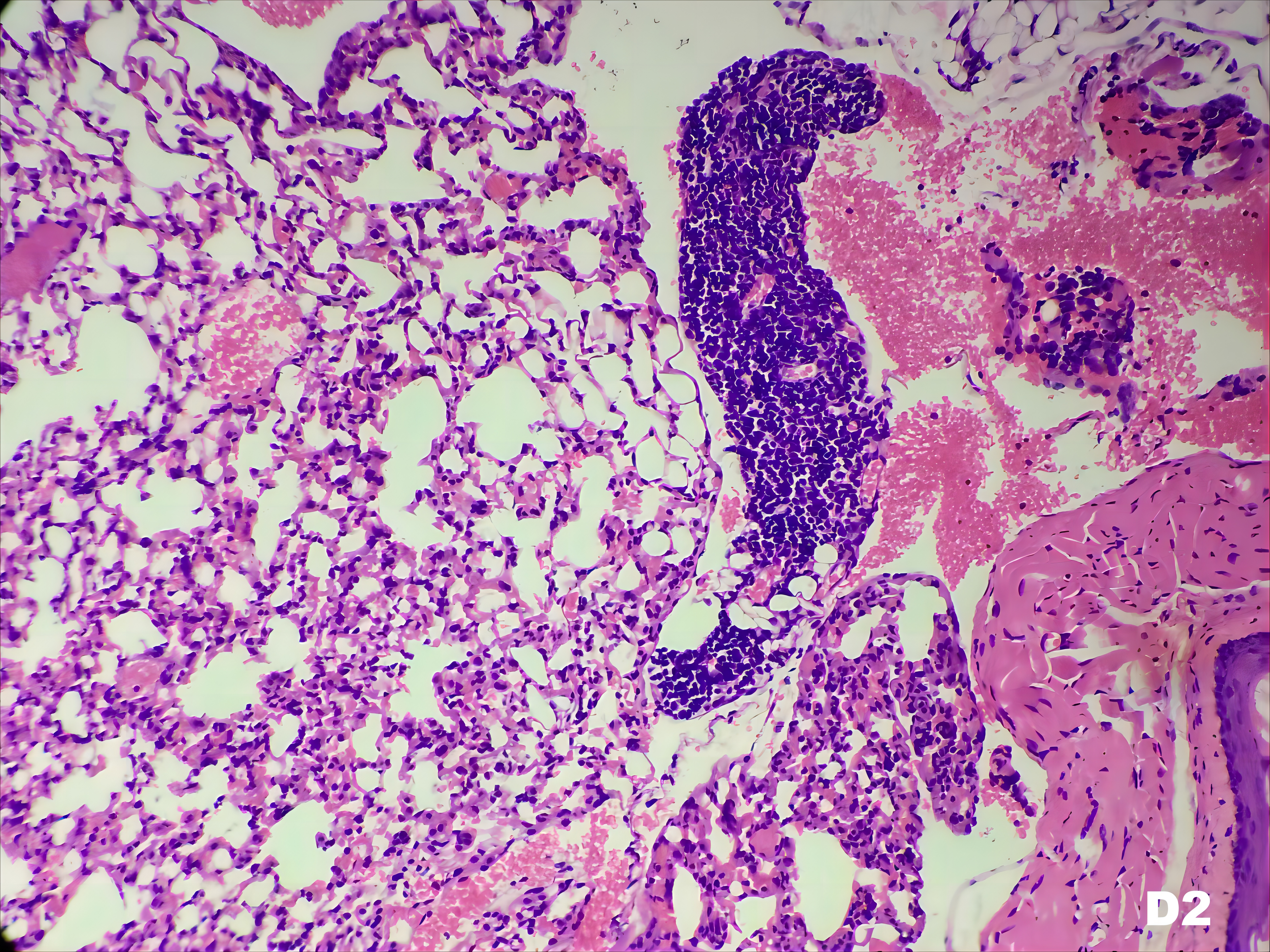

Supplement: Supplementary file 12 — Supplementary file12 (PNG 17099 KB) [file 10482_2023_1883_MOESM12_ESM.png]

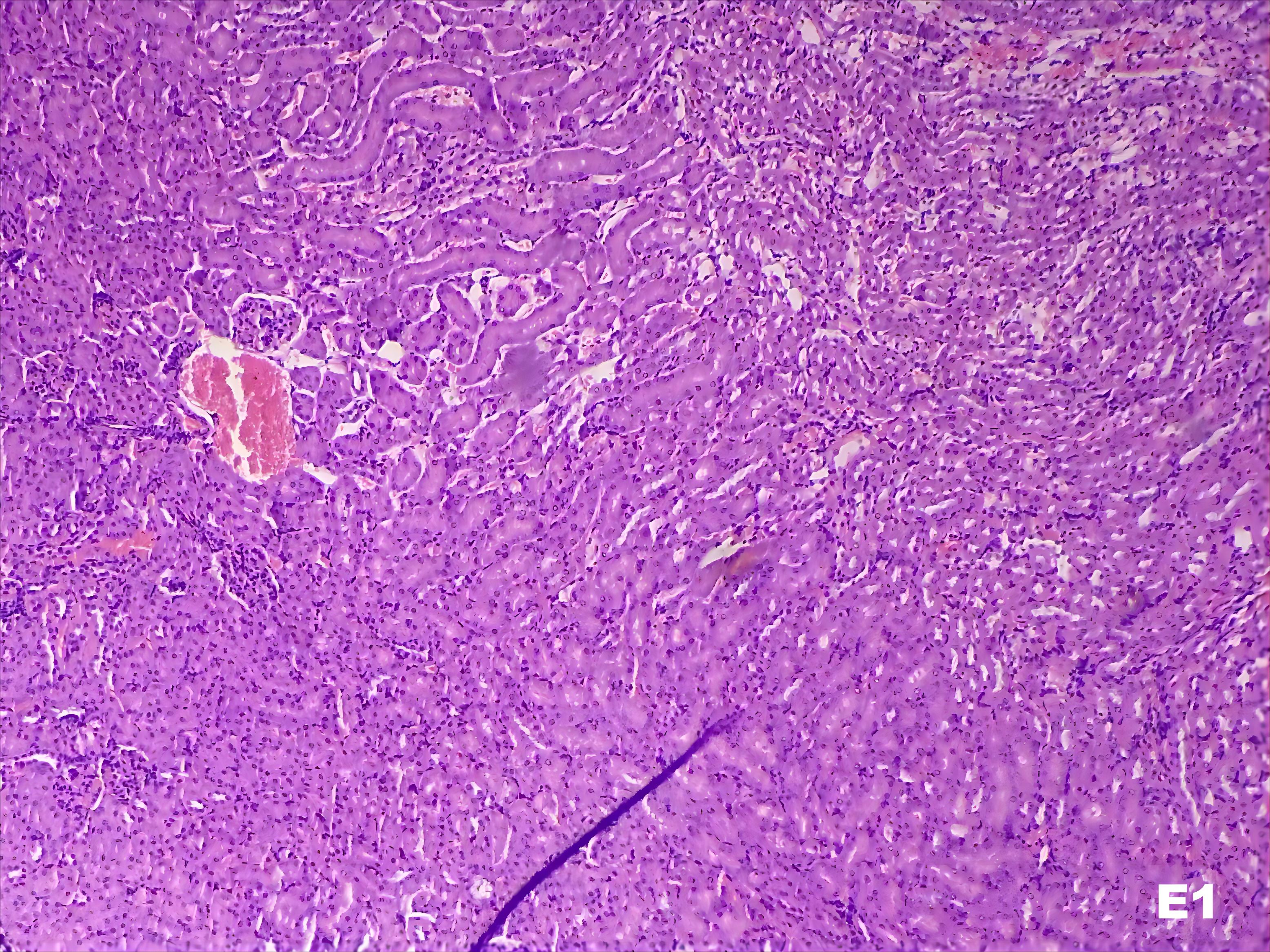

Supplement: Supplementary file 13 — Supplementary file13 (PNG 12843 KB) [file 10482_2023_1883_MOESM13_ESM.png]

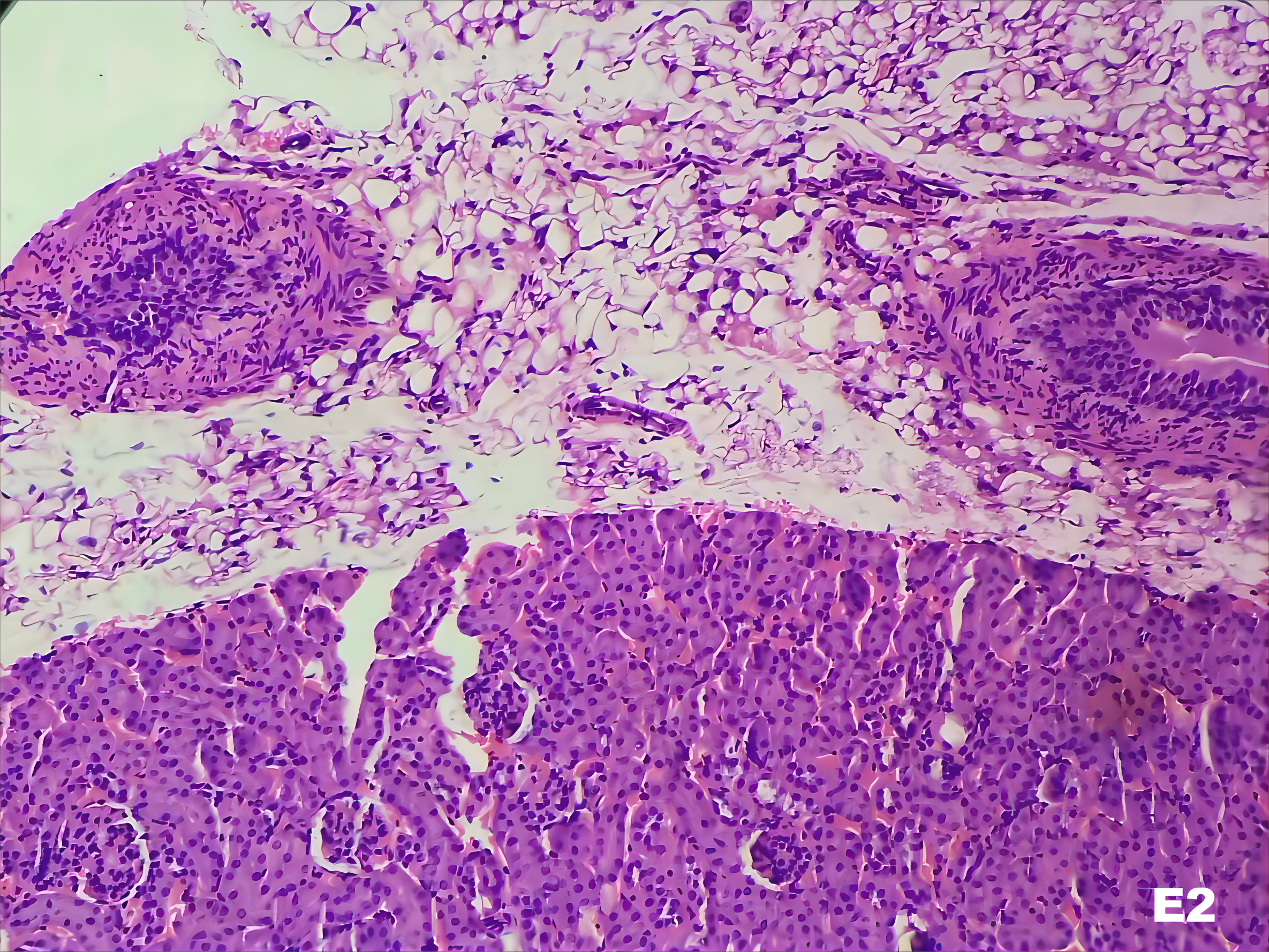

Supplement: Supplementary file 14 — Supplementary file14 (PNG 8248 KB) [file 10482_2023_1883_MOESM14_ESM.png]
